# Frequency of myelin oligodendrocyte glycoprotein antibodies in a large cohort of neurological patients

**DOI:** 10.1177/20552173211022767

**Published:** 2021-06-25

**Authors:** Friederike Held, Sudhakar Reddy Kalluri, Achim Berthele, Ana-Katharina Klein, Markus Reindl, Bernhard Hemmer

**Affiliations:** Department of Neurology, Klinikum rechts der Isar, Medical Faculty, Technische Universität München, Munich, Germany; Clinical Department of Neurology, Medical University of Innsbruck, Innsbruck, Austria; Department of Neurology, Klinikum rechts der Isar, Medical Faculty, Technische Universität München, Munich, Germany; Munich Cluster of Systems Neurology (SyNergy), Munich, Germany

**Keywords:** Myelin-oligodendrocyte glycoprotein, MOG-AD, neuromyelitis optica spectrum disoreders, acute disseminated encephalomyelitis, autoantibody

## Abstract

**Background:**

Myelin oligodendrocyte glycoprotein (MOG) antibody disease (MOG-AD) is recognized as a distinct nosological entity. IgG antibodies against MOG (MOG-Ab) overlap with neuromyelitis optica spectrum disorders (NMOSD) phenotype in adults. However, an increasing number of clinical phenotypes have been reported to be associated with MOG-Ab.

**Objective:**

To investigate the seroprevalence of MOG-Ab under consideration of demographics, disease entities and time course in a large cohort of unselected neurological patients.

**Methods:**

Blood samples of 2.107 consecutive adult neurologic patients admitted to our department between 2016-2017 were tested for MOG-Ab using a cell-based assay. MOG-Ab persistence was analyzed in follow-up samples. External validation was performed in two independent laboratories.

**Results:**

We found MOG-Ab in 25 of 2.107 (1.2%) patients. High antibody ratios were mostly associated with NMOSD and MOG-AD phenotype (5/25). Low ratios occurred in a wide range of neurological diseases, predominantly in other demyelinating CNS diseases (5/25) and stroke (6/25). MOG-Ab persistence over time was not confined to NMOSD and MOG-AD phenotype.

**Conclusion:**

The present study demonstrates the occurrence of MOG-Ab in a wide range of neurological diseases. Only high MOG-Ab ratios were associated with a defined clinical phenotype, but low MOG-Ab ratios were not. The diagnostic value of low MOG-Ab is thus highly limited.

## Introduction

Myelin oligodendrocyte glycoprotein antibody associated disease (MOG-AD) has been recognized as a new disease entity during the last decade. MOG antibodies (MOG-Ab) are found in about 40% of children with first demyelinating event in particular acute demyelinating encephalomyelitis (ADEM).^1–5^ In adults, MOG antibodies are described in about 30-50% of patients suffering from Aquaporin-4 antibody (AQP4-Ab) seronegative Neuromyelitis optica spectrum disorders (NMOSD).^6–11^ Here, NMOSD overlaps with the spectrum of MOG-AD.^11,12^ However, among patients with MOG-AD, only 23% of adults and 31% of children fulfill diagnostic criteria for seronegative NMOSD.^11^ Multiple clinical, radiologic differences exist. In turn, recurrent often bilateral painful optic neuritis (ON) with optic nerve perineuritis and optic disc swelling as well as myelitis of the lower spinal cord are found to be indicative for MOG-AD.^13–17^

By using enzyme linked immunosorbent assay (ELISA) and Westernblot (WB) as detection methods, MOG-Ab have been found in patients with other neurological disorders including multiple sclerosis, stroke and polyneuropathy as well as in viral (HSV, EBV) or autoimmune encephalitis (anti-NMDAR) several years ago. ^10,12,18–22^ To date, detection of MOG-Ab by ELISA, WB or fixed cell-based assay are known to be less specific.^10,23–25^ Live cell-based assay expressing full-length human MOG is the method of choice to specifically detect conformation sensitive, clinically relevant MOG-Ab in pre-selected cohorts.^24,26–28^

However, the overall prevalence of MOG-Ab in an unselected cohort of neurological patients using state of the art detection methods is unknown. In our study we systematically assessed the prevalence of MOG-Ab in adults with neurological disease by using live cell-based assay.

## Methods

### Patient samples

We analyzed sera of 2.107 consecutive neurological patients admitted to in- and outpatient services at the Department of Neurology at the Technical University of Munich between July 2016 and December 2017. The patients were stratified according to the diagnosis at discharge from the hospital. The cohort comprized 13 patients diagnosed with NMOSD/MOG-AD, 282 patients with other demyelinating diseases of the CNS, 584 patients who suffered a stroke and 1.228 patients with a broad range of other neurological diseases. All patients had provided blood samples for research projects. Samples were collected in the biobank of the Department of Neurology, which is part of the Joint Biobank Munich in the framework of the German Biobank Node. Written informed consent was obtained from each participant. The study was approved by the ethics committee of the Technical University of Munich. A small cohort of patients with known MOGAD was included as a reference. Sera of these patients tested MOG-Ab positive in an external laboratory.

### Detection of human MOG antibodies by flow cytometry

Live flow cytometry cell-based assay (CBA-FACS) was performed using LN18 human glioblastoma cell line (LN18) stably transduced with a recombinant plasmid expression vector pLenti6/V5-MOG for full-length human MOG (LN18-MOG) as described previously.^29^ LN18 stably transduced with an empty vector served as a control cell line (LN18-Ctr). To detect MOG-Ab, serum (dilution 1:200) was incubated with LN18-MOG and LN18-Ctr in separated wells followed by fluorochrome-conjugated goat-anti human cross-adsorbed IgG (H + L) (ThermoFisher A-11013, AF 488) (dilution 1:100). Samples were measured on a CytoFLEX S flow cytometry (Beckman Coulter, Brea, USA) and analyzed by using CytExpert software (Beckmann Coulter, Brea, USA). In each experiment, 100 serum samples were analyzed. In each experiment one and the same MOG-Ab positive reference serum was analyzed at different dilutions (1:200 to 1:3200) to monitor the quality of the assay and to allow comparison of data from different experiments. Experiments were discarded if the standard curve with the reference serum was not within the expected range. In a subset of samples MOG-Ab ratios were also analyzed by using a fluorochrome-conjugated goat-anti human IgG (Fc) (ThermoFisher H10120, AF 488) (dilution 1:50) and fluorochrome-conjugated mouse-anti human IgG1 (ThermoFisher A-10631, AF 488) (dilution 1:50) secondary antibody. Cross-reactivity with IgM was also explored (fluorochrome-conjugated goat anti-human IgM, ThermoFisher A-21215, AF 488) (dilution 1:50) in these samples.

### Analysis of MOG Ab positivity

Assessment of antibody binding was conducted by comparing serum antibody binding to LN18-Ctr and LN18-MOG. To quantify antibody binding intensity, median fluorescence intensity (MFI) of the serum staining of LN18-Ctr was subtracted from the staining of LN18-MOG and divided by the MFI of LN18-Ctr to adjust for background staining (CBA ratio; (MFI (LN18-MOG) – MFI (LN18-Ctr))/MFI (LN18-Ctr)).

Because no gold standard for the cut-off of CBA-FACS assays exists, we selected a cut-off for our assay based on the experience with MOG-Ab testing in commercial labs. The binding of the reference sample at a dilution of 1:3200 corresponded to the binding of serum samples that tested weakly positive in a commercial reference lab (Supplemental Material Figure 1).

**Figure 1.**
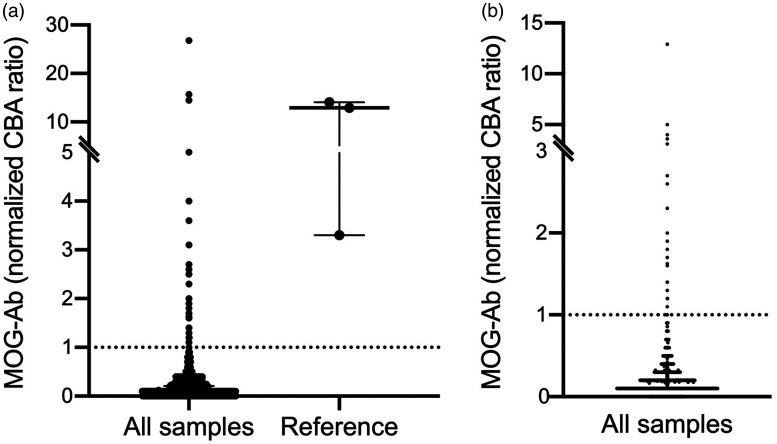
MOG-Ab ratio of all samples and reference. Normalized MOG-Ab ratio of all samples (left column, n = 2.107) and samples from known high MOG-Ab positive patients as a reference (right column, n = 3). Median and interquartile range are expressed (Median (IQR)): *All samples* (0.1 (0.1)), *Reference* (12.9 (10.8)). Values ≥1 indicate MOG-Ab positivity.

Therefore, the cut-off for the presence of MOG-Ab was set to the quotient obtained with the reference sample at a dilution of 1:3200 to compare data obtained from different experiments (normalized CBA ratio; CBA obtained with the test sample/CBA obtained with the reference sample at a dilution of 1:3200). Seropositivity was determined as exceeding this cut-off in at least two quality-proofed assays. Normalized CBA values above 1 indicate the presence of MOG-Ab (Supplemental Material Figure 1). The cut-off for high MOG-Ab in our assay was set to 7.9, which corresponded to a 1:400 dilution of the reference sample. For external validation, coded samples were sent to two independent institutions: Institute 2 (Medical University of Innsbruck, Austria) performed live CBA-IF using transiently transfected HEK 293 cells with a recombinant plasmid expression vector for human full-length human MOG. MOG-IgG was detected using an anti-human IgG (H + L) secondary antibody. The cut-off value for positivity was a titer >1:160 and results were confirmed using an anti-human IgG (Fc) secondary antibody.^27^ Institute 3 was a commercial laboratory, which measured antibodies by fixed CBA-IF using HEK293 cells transiently transfected with a recombinant plasmid expression vector encoding for MOG X11 isoform. MOG-IgG was detected using an anti-human IgG(Fc) secondary antibody. Cut-off value for positivity in the commercial assay was a titer ≥ 1:10 or 1:32.^28^

### Statistics

Normalized CBA MOG-Ab ratios of all tested samples were first described by median and interquartile range (IQR). MOG-Ab positive tested samples of clinically suspected NMOSD (diagnosed according to the International Panel for NMO Diagnosis (IPND) consensus criteria) or MOG-AD and samples of non-NMOSD phenotype were compared by Mann-Whitney non-parametric test.^30^ In a second step, all MOG-Ab positive samples of a non-NMOSD phenotype were stratified in subgroups by entity. Differences of MOG-Ab ratios were analyzed by Kruskal-Wallis and Dunn-Bonferroni Post-hoc-test. An association of gender and MOG-Ab ratio was analyzed using Mann-Whitney Test. Spearman correlation was adjusted to test for an association of age and MOG-Ab ratio. MOG-Ab ratios of follow up courses, if available, were reported with median (IQR). Finally, to quantify interrater-reliability of MOG-Ab ratios (positive vs. negative) between independent laboratories we calculated Cohen’s Kappa. Figures and statistic were generated using GraphPad Prism 8 (GraphPad, San Diego, CA).

## Results

### Prevalence of MOG-Ab in patients with neurological diseases

We tested sera from 2.107 consecutive patients admitted to our hospital for MOG-Ab. Samples were not preselected for diagnoses or risk of MOG-Ab associated disease. The results were compared to those from a small cohort of patients with known MOG-Ab associated disease and known high antibody ratio (Figure 1). We detected MOG-Ab exceeding our cut-off in 25 of 2.107 samples (1.2%). Interestingly the test results were not dichotomized in positive and negative results but rather a continuum spanning from negative to borderline to clearly positive MOG-Ab ratios.

### Characteristics of MOG-Ab positive patients

**Figure 2.**
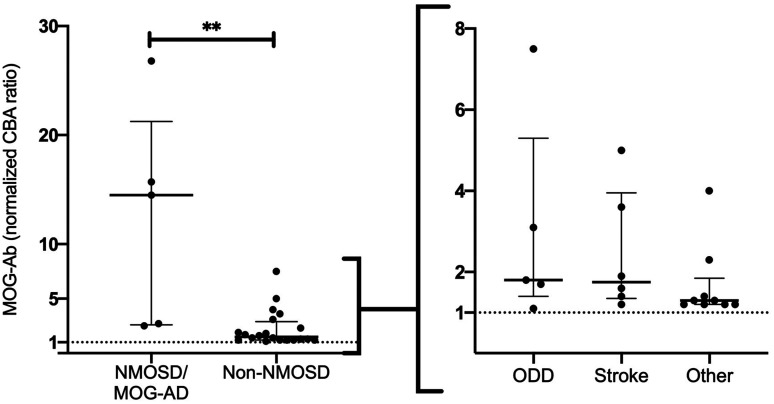
MOG-Ab ratio of subgroups. Samples of positive MOG-Ab ratio (normalized) with NMOSD/MOG-AD (n=5) and Non-NMOSD (n=20) phenotype and further stratified in subgroups by entity: Other demyelinating diseases (ODD) of the CNS (n=5), Stroke (n=6) and Other (n=9). Median and interquartile range are expressed (Median (IQR)): *NMOSD/MOG-AD* (14.5(18.7)), *Non-NMOSD* (1.5(1.7)), *ODD* (1.8(3.9)), *Stroke* (1.8(2.6)), *Other* (1.3(0.7)). Values ≥ 1 indicate MOG-Ab positivity. **p-value 0.0037.

5 of the 25 MOG-Ab positive patients had an NMOSD or MOG-AD phenotype, 5 were diagnosed with other demyelinating diseases of the CNS (ODD), 6 patients with stroke and 9 with a broad spectrum of other neurological diseases (Table 1). High MOG-Ab titer (cut-off ≥7.9) were only seen in patients with a classical NMOSD or MOG-AD phenotype. When we compared antibody ratios among groups, MOG-Ab ratio were significant higher in the NMOSD/MOG-AD group compared to patients with a non-NMOSD phenotype (Median (IQR): NMOSD/MOG-AD (14.5(18.7)), Non-NMOSD (1.5(1.7); p-value 0.0037). No relevant ratio difference was observed between non-NMOSD subgroups (Median (IQR): ODD (1.8(3.9)), Stroke (1.8(2.6)), Other (1.3(0.7)) (Figure 2).

**Table 1.** Characteristics in MOG-Ab positive samples stratified in subgroups.

Diagnose	Sex	Age (y)	MOG-Ab ratio
NMOSD			
MOG-AD	F	26	26.8
MOG-AD	F	39	15.7
NMOSD	F	36	14.5
NMOSD	F	39	2.5
NMOSD	F	35	2.7
Other demyelinating diseases of the CNS			
Isolated optic neuritis	F	48	1.1
Isolated optic neuritis	F	25	3.1
Clinically isolated syndrome with optic neuritis	F	25	1.8
Radiologically isolated syndrome	M	31	1.7
Isolated optic neuritis	F	25	7.5
Cerebral infarct			
Stroke (brain stem)	M	57	1.6
TIA	F	86	1.4
Stroke (MCA)	M	56	3.6
Stroke (PICA)	M	54	5.0
Stroke (MCA)	F	83	1.9
Stroke (MCA)	M	49	1.2
Others			
Cranial nerve palsy	F	65	1.3
Epilepsy	M	67	1.2
Epilepsy	M	69	2.3
Gait disturbance	M	81	1.2
Dystonia	M	47	1.2
Polyneuropathy	F	58	1.2
Infectious Encephalitis	M	77	4.0
Lymphoma	F	58	1.3
Infectious Encephalitis	F	85	1.4

TIA: transient ischemic attack; MCA: middle cerebral artery; PICA: posterior inferior cerebral artery; F: female; M: male; Y: years.

Note: MOG-Ab are displayed as normalized CBA ratio.

Overall gender was significantly associated with MOG-Ab ratio (p = 0.04) with higher ratios in female. In accordance, we observed a female predominance (63%) in MOG-Ab positive patients, which was most pronounced in the NMOSD/MOG-AD and the ODD groups. By contrast, a male predominance was seen in stroke and other neurological disease groups (Table 2), the majority of whom had low or borderline MOG-Ab ratios. Interestingly groups also differed with respect to age, but the difference did not reach significance (r = −0.3208). MOG-Ab positive NMOSD, MOG-AD and ODD patients were younger than MOG-Ab positive patients in the stroke and other neurological diseases groups.

**Table 2.** Demographic aspects of subgroups.

Characteristics	NMOSD/MOG-AD	ODD	Stroke	Others
Gender F:M	5:0	4:1	1:2	4:5
Age mean (range)	35 (26–39)	31 (25–48)	64 (49–86)	67 (47–85)
Median MOG-Ab (interquartile range)	14.5 (18.7)	1.8 (3.9)	1.8 (2.6)	1.3 (0.7)

F: female; M: male; ODD: other demyelinating disease.

Note: MOG-Ab ratios are displayed as normalized CBA ratio.

### Longitudinal analysis of MOG-Ab ratio

**Figure 3.**
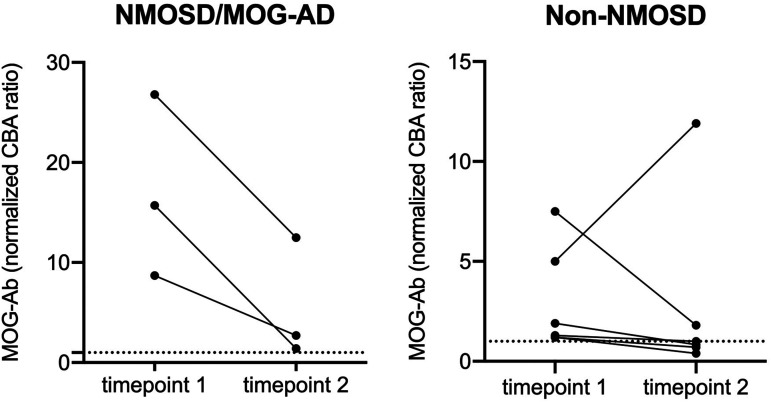
Follow up data of MOG-Ab positive sample. Normalized MOG-Ab ratio at two different timepoints are expressed according to NMOSD/MOG-AD (n=3) and Non-NMOSD (n=6) phenotype. Median and interquartile ranges for all subgroups and timepoints (Median (IQR)): *NMOSD* timepoint one = 15.70 (18.1); timepoint two = 2.7 (11.1); *Non-NMOSD* = timepoint one= 1.6 (4.4); timepoint two = 0.9 (3.7).

Follow-up analyzes were conducted of MOG-Ab positive samples if available (Figure 3). Timepoint one indicates the timepoint at admission to the hospital, in most cases due to an acute disease event (e.g. relapse, stroke, seizure) while samples at timepoint two were collected during scheduled follow up consultation. Median time to follow up was 26 months. Except for one patient, MOG-Ab ratios were lower at follow up consultation. Persistent MOG-Ab were observed in all disease groups: none of the NMOSD/MOG-AD patients become MOG-Ab negative during follow up compared to 50% of patients with a non-NMOSD phenotype (Median (IQR): NMOSD/MOG-AD timepoint one = 15.70 (18.1); timepoint two = 2.7 (11.1); Non-NMOSD = timepoint one = 1.6 (4.4); timepoint two = 0.9 (3.7)). All MOG-Ab positive NMOSD/MOG-AD patients had a relapse at timepoint one and were relapse-free during follow-up period, two of them under maintenance immunosuppressive therapy with rituximab (Supplemental Material Table 1).

### Internal and external validation

We performed external validation of our results in two different laboratories using either live or fixed CBA-IF. Most samples that tested positive in our assay and some that were tested positive in external laboratories but tested negative in our assays were also analyzed. External institutes were blinded for clinical information and MOG-Ab ratio tested in our laboratory.

Samples of patients with NMOSD/MOG-AD phenotype and high MOG-Ab ratio tested positive in all three laboratories. Most samples with low ratios, independent of the patient’s diagnosis, also tested positive in laboratory 2 while half of the patients tested negative in laboratory 3. Interestingly, some of the samples that were negative in our laboratory (laboratory 1) and laboratory 2 tested positive in laboratory 3 (Supplemental Material Table 2). Concordance of tested MOG-Ab ratio was high between laboratory 1 and 2 (kappa: 0.679), moderate between laboratory 2 and 3 (kappa: 0.406) and only low between laboratory 1 and 3 (kappa: 0.214).

A cut-off value indicating clinically suspicious MOG-Ab positivity was not derivable throughout all three institutes without missing positive samples, as ratio- and titer-concentrations differed at lower levels.

To investigate whether differences between laboratories were caused by different secondary antibodies used for detection, we analyzed the samples by our life CBA using anti IgG-Fc, IgG1 and IgM secondary antibodies. We found no cross-reactivity of MOG-IgG positive tested samples with anti IgM. Only one sample that was tested negative with each anti IgG secondary antibody, was tested positive for IgM (data not shown). Concordance of the test results for anti IgG H + L, IgG Fc and IgG1 was high for high MOG-Ab ratios in patients with an NMOSD/MOG-AD phenotype (Supplemental Material Table 2). In patients with other demyelinating or noninflammatory diseases, differences were observed between MOG-Ab ratios obtained with different secondary antibodies. The correlation of test results was best between our live CBA using IgG H + L and IgG-Fc and the life CBA of laboratory 2 using IgG-Fc as secondary antibody. None of the secondary antibodies seemed to be superior in reducing the rate of MOG-Ab positive samples of patients with non-inflammatory diseases.

## Discussion

In a cohort of 2.107 adult patients admitted to a Level I university neurology department 1,2% were MOG-Ab positive. The distribution of MOG-Ab ratio was not dichotomized but rather a continuum between negative, borderline and positive samples. MOG-Ab ratios were highest in patients suffering from NMOSD/MOG-AD compared to non-NMOSD phenotype. Younger age and female gender were associated with a higher MOG-Ab ratio. Follow up examination revealed a decrease of MOG-Ab at follow up in most cases. Whereas all NMOSD/MOG-AD patients remained MOG-Ab positive during follow up, 50% of patients with a non-NMOSD/MOG-AD phenotype became MOG-Ab negative. For external validation multicenter testing revealed high consistency for high MOG-Ab ratios related to NMOSD/MOG-AD phenotype whereas the results differed for low MOG-Ab ratios between laboratories. Results were more consistent between the laboratories with tests based on live CBA compared to fixed CBA. Regardless of the type of secondary antibody used for the detection, MOG-Ab were found in a small subset of patients with non-inflammatory neurological diseases.

The implementation of CBA to measure antibodies targeting full-length human MOG expressed in mammalian cells enables a more specific detection of clinically relevant antibodies.^23–25,27,28^ Assay sensitivity and specificity increased over time by knowing the effect of serum dilution, impact of IgG-specific secondary antibody and the reading methods like FACS or IF.^24,26^ In turn, MOG-Ab are only rarely found in other demyelinating diseases of the CNS like MS and should give rise to question the diagnose.^31,32^ However, the decision whether antibody titer is false positive or not is not always easy, especially if MOG-Ab status using CBA differs between institutes as it has been the case in recent multicenter studies.^27,28^ An optimal assay cut-off value still not exist and whereas the clinical relevance of high MOG-Ab titer is widely accepted, the interpretation of low and borderline titer is still uncertain.^10,25^ This coincided with our data detecting low MOG-Ab ratio in a wide range of neurological diseases and highest MOG-Ab ratio in NMOSD/MOG-AD. Of these, most were female and relatively young at onset. Previous studies came to partly contradictory statements: also a balanced gender distribution or predominance of male gender was reported.^1,17,33–35^ We suppose that the association of age and level of MOG-Ab ratio is due to manifestation of autoimmune diseases in younger years and occurrence of stroke in older than directly associated with each other. Whether the level of MOG-Ab titer is predictive for the course of disease has not yet been finally clarified. Higher MOG-Ab titer during relapse than remission but also contradictory findings with low titers during relapse and high titers during remission have been reported.^12,23,34,36^ Decreasing titer are usually found in monophasic disease courses and conversion to negativity was reported to be associated with no further relapses.^12,33,37^ However, relapses were occasionally observed in seronegative adult patients so that the predictive value of the antibody titer is limited with regard to the course of disease.^38–40^ We found higher MOG-Ab ratios at timepoint one (during relapse) than at timepoint two (scheduled follow-up in remission) in NMOSD/MOG-AD patients. Two of these patients receive maintenance immunosuppressive therapy due to severe index event. The other patient has no maintenance immunosuppressive therapy. All of these patients were relapse free during follow-up despite persistent detection of MOG-Ab over several years.

Fluctuation of MOG-Ab titer and often short observation time complicate the predictive value.^38^ So far follow up examination of MOG-Ab titers in diseases distinct to NMOSD have not yet been conducted. Here we demonstrated persistent MOG-Ab also in few patients analyzed in our study. However, 50% become seronegative which emphasizes the occurrence of MOG-Ab also as a bystander phenomenon in borderline and thus false-positive tested samples.

To further evaluate the results obtained by our assay, we re-tested a selection of samples by using anti IgG Fc, IgG1 and IgM secondary antibody and carried out validation in two independent external laboratories. The findings were rather consistent for patients with high MOG-Ab. The type of CBA (fixed or live) and secondary antibody used had no impact in these patients. However, ratio and titers differed between live and fixed CBA and used IgG secondary antibody for negative and borderline patients. Waters et al. determined high cross-reactivity of IgG H + L to IgM.^26^ By using a multicross-adsorbed IgG H + L, we observed no cross-reactivity with IgM in our assay. Only one patient was MOG-IgM positive but tested negative with all three secondary IgG antibodies.

Recent multicenter comparison revealed live CBA as the gold standard to detect MOG-Ab.^27,28,41,42^ IgG Fc and IgG1 secondary antibody are considered equally in regard to specificity and thus recommended to detect MOG-Ab.^23,43^ The higher consistency was observed in our study between our laboratory and laboratory 2, which both use live-CBA. Independent of the secondary antibody used, MOG-Ab were detected in a small proportion of patients with non-inflammatory diseases. None of the secondary antibodies seemed to be superior with respect to sensitivity and specificity suggesting that preadsorbed anti IgG H + L secondary antibodies are also suitable for screening for MOG-Ab.

The distribution of MOG-Ab in this large cohort of non-preselected patients demonstrates the difficulties of interpreting the results of MOG-Ab testing. The distribution between positive and negative tests is not dichotomized but rather a continuum with no obvious cut-off. This observation in combination with the inconsistency observed between laboratories for low and borderline MOG-Ab ratios, even by using same methods of testing, is important for use of MOG-Ab testing in clinical practice. Currently used live CBA showed excellence agreement for clearly positive and negative samples in recent multicenter testing, whereas fixed CBA did not.^27^ High MOG-Ab titers can be reliably measured and are certainly helpful to establish the disease in patients with phenotypic features of MOG-AD. However, low and borderline MOG-Ab titers need to be interpreted with caution especially in patients without a classical clinical phenotype. Re-testing should be considered if MOG-AD is suspected as the concentration of MOG-Ab often depends on disease activity, fluctuating titers are known and assay specificity varied. ^23,38,44^

Our study has some limitations: first of all, the sample size of subpopulations is very small. Subgroup analyses are therefore only of limited significance. The same applies to follow-up analyses. We had to define an individual cut-off value for MOG-Ab positivity due to the lack of a validated detection method. Methodological differences of CBA lead to limited comparability of MOG-Ab titer of institute 3 with institute 2 and our laboratory. Furthermore, screening for a rare biomarker in a large unselected cohort implicates false positive results.

In summary the clinical relevance of MOG-Ab seems to be titer dependent. High MOG-Ab titers are likely specific for a MOG-AD phenotype and thus helpful for diagnostic purposes. The diagnostic value of low and borderline MOG-Ab titer is highly limited. To avoid false-positive results, MOG-Ab testing should be limited to patients with a clinical phenotype compatible with MOG-AD.

## Supplemental Material

sj-pdf-1-mso-10.1177_20552173211022767 - Supplemental material for Frequency of myelin oligodendrocyte glycoprotein antibodies in a large cohort of neurological patients Click here for additional data file. Supplemental material, sj-pdf-1-mso-10.1177_20552173211022767 for Frequency of myelin oligodendrocyte glycoprotein antibodies in a large cohort of neurological patients by Friederike Held, Sudhakar Reddy Kalluri, Achim Berthele, Ana-Katharina Klein, Markus Reindl and Bernhard Hemmer in Multiple Sclerosis Journal – Experimental, Translational and Clinical
