# Supplementary material for: Frequency of myelin oligodendrocyte glycoprotein antibodies in a large cohort of neurological patients
Source: Mult Scler J Exp Transl Clin. 2021 Jun 25;7(2):20552173211022767. doi: 10.1177/20552173211022767 (PMC8246507; doi:10.1177/20552173211022767)
Supplement: sj-pdf-1-mso-10.1177_20552173211022767 - Supplemental material for Frequency of myelin oligodendrocyte glycoprotein antibodies in a large cohort of neurological patients [file sj-pdf-1-mso-10.1177_20552173211022767.pdf]

Supplementary Appendix

Supplementary Figure 1: Analyzation of MOG-Ab positivity in CBA-FACS

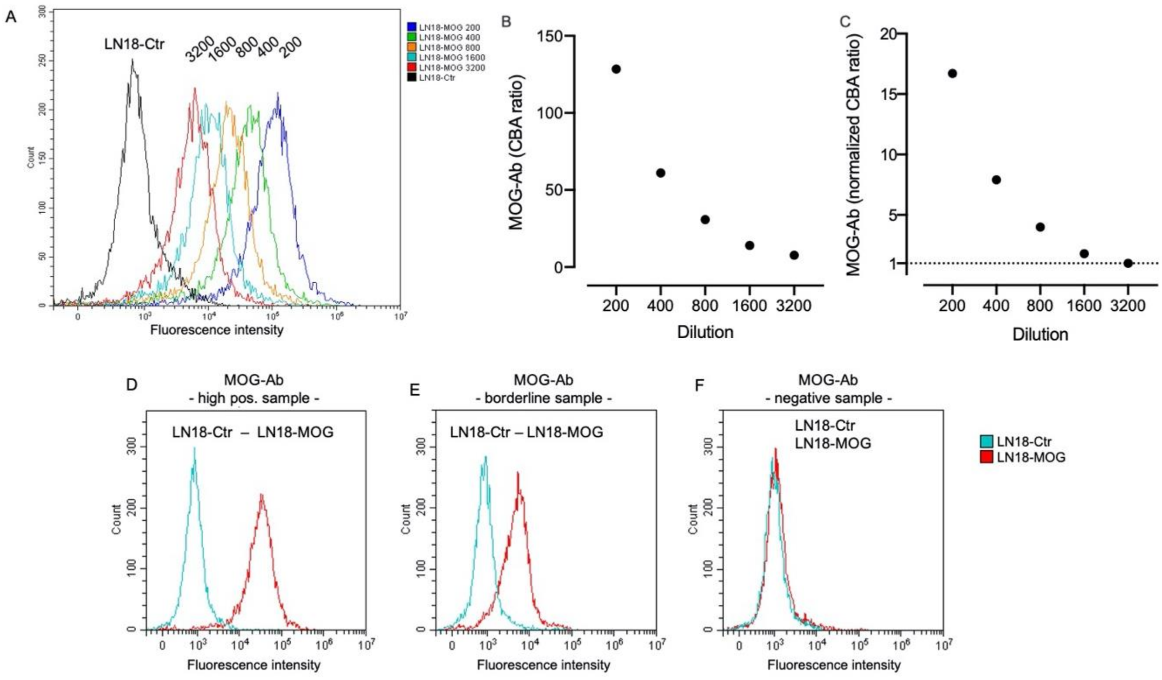

Figure 1: Assessment of MOG-Ab positivity by flow cytometry live cell based assay. *A*: MFI of control cell line transduced with an empty vector (LN18-Ctr) and MOG-expressing cells (LN18-MOG) in a dilution series (1:200 – 1:3200) using anti-IgG H+L as secondary antibody. Fluorescence intensity indicates Fluochrom-conjugated anti human MOG-IgG binding to MOG. *B:* Standard curve of MOG-Ab ratios of a reference sample determined in CBA-FACS (CBA ratio at a dilution of 1:3.200 (7.7); 1:1.600 (14.0); 1:800 (30.8); 1:400 (61.0); 1:200 (128.4)). *C:* Normalized CBA-Ratio of a reference sample in a dilution series with 1 at a dilution of 1:3200 set as the cut-off for MOG-Ab positivity (normalized CBA-Ratio at a dilution of 1:3.200 (1.0); 1:1.600 (1.8); 1:800 (4.0); 1:400 (7.9); 1:200 (16.7)). Ratios above 7.9, which correspond to a 1:400 dilution of the reference sample, were considered high antibody titer. *D-F:* MFI of control cell line and MOG-Ab high-positive, borderline and negative sample respectively. MFI: median fluorescence intensity.

Supplementary Table 1: Follow-up Analyses of MOG-Ab positive tested samples

Diagnose	First sample		Follow-up sample
	MOG-Ab ratio	Intervall	MOG-Ab ratio
**NMOSD/MOG-AD**			
MOG-AD	26.8	470	12.5
MOG-AD	15.7	797	1.4
NMOSD	8.7	659	2.7
**Other demyelinating diseases**			
Isolated optic neuritis	7.5	353	1.8
**Cerebral Infarct**			
Stroke (PICA)	5	1067	11.9
Stroke (MCA)	1.9	539	0.84
Stroke (MCA)	1.2	1100	0.7
**Others**			
Cranial nerve palsy	1.3	1260	1
Polyneuropathy	1.2	1196	0.4

Abbreviations: MCA= Middle cerebral artery; PICA= Posterior inferior cerebellar artery. Interval specified in days. MOG-Ab ratios are displayed as normalized CBA ratio.

Supplementary Table 2: External validation of MOG-Ab ratio

Diagnose	MOG-Ab				
	Lab1			Lab 2	Lab 3
**NMOSD/MOG-AD**	**IgG H+L**	**IgG Fc**	**IgG1**		
MOG-AD	pos (26.8)	pos (43.5)	pos (389.06)	pos (1:5.120)	pos (1:3.200)
MOG-AD	pos (15.7)	pos (1.61)	pos (5.39)	low pos (1:160)	pos (1:32)
NMOSD	pos (14.5)	pos (13.07)	pos (86.77)	pos (1:1.280)	pos (1:100)
NMOSD	pos (2.7)	pos (11.87)	pos (61.27)	low pos (1:320)	low pos (1:10)
NMOSD	pos (2.5)	pos (2.25)	pos (1.08)	low pos (1:160)	pos (1:100)
**Other demyelinating diseases of the CNS**					
Isolated optic neuritis	pos (7.5)	pos (15.01)	pos (89.31)	pos (1:1280)	pos (1:320)
Isolated optic neuritis	pos (3.1)	pos (2.73)	pos (1.66)	low pos (1:320)	low pos (1:10)
Clinically isolated syndrome with optic neuritis	pos (1.8)	pos (4.11)	pos (8.23)	low pos (1:320)	low pos (1:10)
Radiologically isolated syndrome	pos (1.7)	pos (1.89)	pos (2.52)	neg	neg
Isolated optic neuritis	pos (1.1)	pos (1.89)	neg	low pos (1:160)	neg
Isolated Myelitis	neg	neg	pos (1.15)	neg	pos (1:40)
Multiple Sclerosis	neg	neg	neg	neg	neg
Clinically isolated syndrome with optic neuritis	neg	pos (1.03)	neg	neg	pos (1:32)
Isolated optic neuritis	neg	no sample	no sample	neg	pos (1:40)
**Cerebral Infarct**					
Stroke (PICA)	pos (5.0)	pos (7.07)	neg	low pos (1:320)	neg
Stroke (MCA)	pos (3.6)	no sample	no sample	neg	pos (1:32)
Stroke (MCA)	pos (1.9)	pos (3.23)	neg	neg	neg
Stroke (brain stem)	pos (1.6)	pos (2.29)	pos (3.14)	neg	neg
TIA	pos (1.4)	pos (2.08)	pos (9.46)	low pos (1:160)	pos (1:32)
Stroke (MCA)	pos (1.2)	pos (1.35)	neg	low pos (1:160)	neg
**Others**					
Infectious Encephalitis	pos (4.0)	pos (3.47)	pos (10.31)	low pos (1:320)	neg
Epilepsia	pos (2.3)	pos (1.96)	neg	low pos (1:160)	neg
Infectious Encephalitis	pos (1.4)	neg	neg	neg	neg
Cranial nerve palsy	pos (1.3)	pos (1.05)	neg	low pos (1:160)	pos (1:32)
Lymphoma	pos (1.3)	neg	pos (1.02)	low pos (1:320)	neg
Gait disturbance	pos (1.2)	neg	neg	low pos (1:160)	low pos (1:10)
Dystonia	pos (1.2)	pos (1.46)	pos (7.76)	low pos (1:320)	low pos (1:10)
Polyneuropathy	pos (1.2)	pos (1.74)	pos (4.66)	low pos (1:160)	pos (1:32)
Epilepsia	pos (1.2)	neg	neg	low pos (1:160)	low pos (1:10)
Paroxysmal sensation	neg	neg	neg	neg	neg
Transient hemianopsia	neg	neg	neg	neg	neg
Urinary incontinence	neg	neg	pos (1.14)	neg	neg
Polyneuropathy	neg	neg	neg	neg	neg
Dystonia	neg	neg	neg	neg	neg

Samples ordered by level of MOG-Ab ratio by using IgG H+L in Laboratory 1. Cut off values for MOG-Ab positivity of the respective laboratories: Lab 1 MOG-Ab ratio (IgG H+L) ≥1 positive; Lab 2 MOG-Ab titer ≥ 1:160 low positive, ≥ 1:640 positive; Lab 3: MOG-Ab titer ≥1:10 low positive, ≥1:32 positive. There was lack of material of two samples for re-testing ("no sample").

Abbreviations: PICA: Posterior inferior cerebellar artery; MCA= Middle cerebral artery; TIA= Transient ischemic attack; PNP= Polyneuropathy. The interpretation of the test result and the ratio and titer (shown in parenthesis for positive samples) are displayed. MOG-Ab in laboratory 1 are displayed as normalized CBA ratio.

Supplementary Table 3: Diagnoses of patient cohort (according to ICD-10-GM)

	**Patient Diagnoses (ICD-10-GM)**
1	A69.2
2	A81.2
3	A84.1+G05.1
4	A86
5	A87.9
6	B00.4+G05.1
7	B01.0
8	B01.1+G05.1
9	B02.2+G05.1
10	B45.1+G02.1
11	C70.0
12	C71.8
13	C79.3
14	C83.3
15	D86.8+53.2
16	E51.2
17	E56.8
18	E75.2
19	F01.2
20	F05.0
21	F44.5
22	G00.1
23	G00.9
24	G02.0
25	G02.1
26	G03.2
27	G04.8
28	G12.1
29	G12.2
30	G13.1
31	G20.1-
32	G20.2-
33	G23.0
34	G23.1
35	G23.2
36	G23.3
37	G24.8
38	G25.81
39	G31.81
40	G35.0-
41	G35.1-

42	G35.2-
43	G35.3-
44	G36.0
45	G36.8
46	G37.7
47	G37.8
48	G40.0
49	G41.0
50	G43.1
51	G43.2
52	G44.0
53	G44.2
54	G44.8
55	G45.3-
56	G45.4-
57	G45.9
58	G50.1
59	G51.0
60	G52.7
61	G57.2
62	G58.0
63	G59.0
64	G61.0
65	G61.8
66	G62.9
67	G70.0
68	G81.0
69	G81.1
70	G82.5-
71	G83.49
72	G90.2
73	G90.5.-
74	G91.29
75	G93.2
76	G99.2
77	H02.4
78	H34.1
79	H46.0
80	H47.0
81	H47.1
82	H48.1
83	H49.0
84	H49.1
85	H49.2

86	H49.4
87	H51.2
88	H53.2
89	H53.4
90	H81.1
91	H81.2
92	I60.1
93	I61.0
94	I61.4
95	I63.0
96	I63.1
97	I63.3
98	I63.4
99	I63.8
100	I67.6
101	I67.7
102	I68.2
103	I72.0
104	I72.6
105	M48.02
106	M54.1-
107	M79.70
108	N39.42
109	R11
110	R20.1
111	R26.1
112	R27.0
113	R40.0
114	R41.2
115	R42
116	S14.3
117	S44.2
